# Specific Items of Enhanced Recovery After Surgery for Liver Surgery in Cirrhotic Patients: A Systematic Review

**DOI:** 10.1002/wjs.12677

**Published:** 2025-06-23

**Authors:** Gaëtan‐Romain Joliat, Constant Delabays, Emilie Uldry, Valérie Addor, David Fuks, Emmanuel Melloul

**Affiliations:** ^1^ Department of Visceral Surgery Lausanne University Hospital CHUV University of Lausanne (UNIL) Lausanne Switzerland; ^2^ Department of Development and External Affairs Lausanne University Hospital CHUV Lausanne Switzerland

**Keywords:** cirrhosis, hepatectomy, hepatic, rehabilitation

## Abstract

**Background:**

Enhanced Recovery After Surgery (ERAS) pathways have been shown to be safe in patients undergoing hepatectomy. Due to cirrhosis‐induced complications, specific or additional perioperative items might need to be implemented. This study systematically reviewed the literature to assess specific items to be included in future ERAS protocol for the perioperative management of cirrhotic patients undergoing hepatectomy.

**Methods:**

A systematic review was performed until September 2024. Evaluated items were prevention and management of perioperative ascites, encephalopathy prevention, perioperative anticoagulation, perioperative nutrition, prophylactic abdominal drainage, postoperative analgesia, vascular filling, and prevention of liver failure. Levels of evidence were given for each specific item based on the Grading of Recommendations, Assessment, Development and Evaluations (GRADE) system.

**Results:**

A total of 3123 articles were screened, and 81 articles were included in the final analysis of the eight evaluated items. Five items had a moderate or high level of evidence. Summary of the five items with moderate/high evidence level were (i) preoperative ascites should be controlled before surgery, (ii) mechanical and pharmacological venous thromboembolism prophylaxis is recommended postoperatively if coagulation tests are within normal range, (iii) systematic drainage among patients with cirrhosis is not recommended, (iv) acetate‐buffered solutions should be preferred over Hartmann solutions or 0.9% saline, and (v) portal hypertension should be optimized preoperatively, and minimal invasive surgery should be favored to decrease liver failure rates.

**Conclusion:**

This systematic review comprehensively evaluated specific perioperative items for cirrhotic patients undergoing liver surgery. Level of current evidence remains moderate, and future research are still needed to develop specific recommendations for ERAS in cirrhotic patients undergoing liver surgery.

## Graphical abstract

Enhanced Recovery After Surgery (ERAS) pathways have been shown to be safe in patients undergoing hepatectomy. Due to cirrhosis‐induced complications, specific or additional perioperative items might need to be implemented. This study systematically reviewed the literature to assess specific items to be included in future ERAS protocol for the perioperative management of cirrhotic patients undergoing hepatectomy. A systematic review was performed until September 2024.

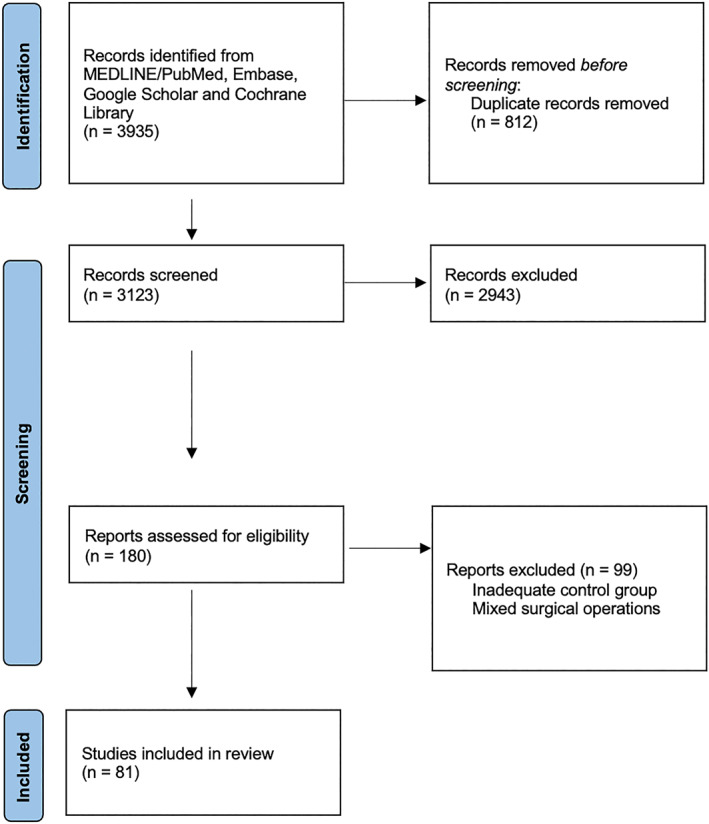

## 1 Introduction

Enhanced Recovery After Surgery (ERAS) has become the standard of care in various surgery types due to its ability to reduce morbidity, shorten length of stay (LoS), and lower costs, all while having no negative impact on readmission rates or mortality [1, 2, 3, 4, 5, 6]. This also applies to liver surgery, with several meta‐analyses recently showing identical benefits [7, 8, 9].

ERAS guidelines for liver surgery were recently updated [10, 11]. These guidelines are mainly based on studies on noncirrhotic patients. Cirrhosis has systemic impact, affecting fluid balance, coagulation, immune function, nutritional status, and leads to dysfunction in multiple organs. Patient with liver cirrhosis are particularly vulnerable to the stress caused by surgery and anesthesia [12]. Those changes induce higher postoperative morbidity and mortality, especially following liver surgery [13, 14, 15]. A recently published meta‐analysis based on five comparative studies found that ERAS for liver surgery in cirrhotic patients was safe and decreased postoperative complications and LoS [16]. Due to the unique pathophysiological changes in cirrhosis, preoperative optimization is paramount and some ERAS items could be adapted and refined to better suit the specific needs of cirrhotic patients.

The present systematic review aimed to evaluate the current literature on liver surgery in cirrhotic patients and identify specific preoperative, intraoperative, and postoperative ERAS items that may need to be refined to improve cirrhotic patient outcome after hepatectomy.

## 2 Material and Methods

### 2.1 Item Selection

Following the ERAS guidelines update and publication of a recent meta‐analysis on ERAS in cirrhotic patients, items associated with thrombosis prophylaxis, perioperative nutrition, abdominal drainage, pain management, and vascular filling were often mentioned in the included articles as potential elements to be adapted and tailored in case of cirrhotic patients undergoing hepatectomy [11, 16]. Moreover, several articles underlined the specificities of ascites and encephalopathy in cirrhotic patients and their impact on the perioperative management in liver surgery [17, 18, 19, 20, 21]. Therefore, eight items that could require specific management in cirrhotic patients were selected beforehand based on the abovementioned criteria by the three main authors (GRJ, CD, and EM) to undergo a systematic literature search: prevention/management of perioperative ascites, encephalopathy prevention, perioperative anticoagulation, perioperative nutrition, prophylactic abdominal drainage, postoperative analgesia, vascular filling, and posthepatectomy liver failure (PHLF) prevention.

A systematic review was conducted to find the most up‐to‐date evidence for cirrhotic patients undergoing hepatectomy. Data were reported following the PRISMA guidelines.

### 2.2 Search

The literature search was performed until September 30, 2024. A systematic review was carried out using the MEDLINE/PubMed, Embase, Google Scholar, and Cochrane Library. The search terms (MeSH and free text) and equations used for each item can be found in the Supporting Information S1. Two authors (GRJ and CD) individually performed the search and preselected the studies to include and evaluate. Article inclusion was then confirmed and validated by the last author.

### 2.3 Eligibility Criteria

Retrospective and prospective studies, reviews, and meta‐analyses were eligible. Case reports or series were excluded. Studies on noncirrhotic patients were excluded. Studies published between January 1, 2000 and September 30, 2024 were considered.

### 2.4 Data Presentation, Quality Assessment, and Statistics

Items were presented individually with objective summary of the literature search. Level of evidence was defined based on the Grading of Recommendations, Assessment, Development and Evaluations (GRADE) system (low/moderate/high). Data from RCT had a high certainty in evidence and were downgraded in case of risk of bias, imprecision, or inconsistency. On the contrary, data from observational studies were classified as low evidence and potentially upgraded based on a large effect magnitude, a clear dose–response gradient, and the impact of residual confounding on magnitude effect. Level of evidence was reported for each item at the result summary end. As several study designs were included in this systematic review, use of different quality scores was required. The quality of each included article was evaluated using the Jadad score (RCT) [22], the Newcastle–Ottawa scale (retrospective comparative studies) [23], the STROBE guidelines (cohort studies) [24], or the AMSTAR checklist (systematic reviews and meta‐analyses) [25]. No statistical analysis was used as no meta‐analysis was performed.

## 3 Results

**FIGURE 1:**
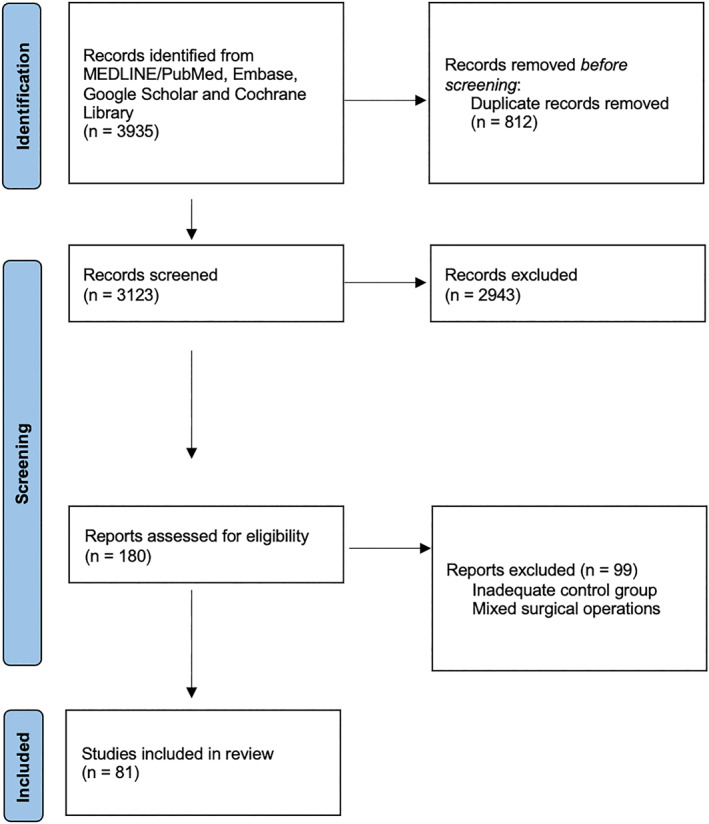
PRISMA flowchart of the systematic review.

3123 articles were screened. After screening and eligibility assessment, 81 articles were included (Figure 1). Table 1 summarizes all included articles. Quality of the included studies is evaluated in Table 2. Table 3 summarizes the eight items.

**TABLE 1:** Summary of included articles.

First author	Publication year	Study type	*N*	Results regarding the concerned item
Ascites				
Zhang	2009	Retrospective cohort	412	Ascites > 500 mL associated with increased morbidity after liver resection among patients with HBV‐related cirrhosis.
Gines	2004	Review	N/A	Preoperative ascites should be controlled with multimodal measure such as low‐sodium diet, diuretics, and paracentesis.
Zheng	2020	Retrospective comparative study	162	ERAS significantly reduces postoperative ascites in cirrhotic patients undergoing hepatectomy compared to non‐ERAS patients (4% vs. 15%, *p* = 0.046).
Chen	2012	Retrospective cohort	73	Indocyanine green retention at 15 min > 10%, tumor size > 10 cm, and red blood cell transfusion are independent risk factors for ascites after liver resection.
Itoh	2017	Retrospective cohort	73	Segment VII resection is a risk factor for refractory ascites (OR 11.8, 95% CI 1.8–76.7, and *p* = 0.009).
Coletta	2020	MA	1321	Laparoscopic liver resection for HCC is associated with lower ascites rate compared to open resection (2.7% vs. 12.9%, *p* < 0.001).
Xu	2018	Retrospective comparative study	1033	Incidence of ascites is significantly lower among patient undergoing laparoscopy (9.4% vs. 31.3%, *p* = 0.030).
Kanazawa	2013	Retrospective comparative study	56	Laparoscopic liver resection significantly decreases the rate of ascites compared to laparotomy (10% vs. 64%, *p* < 0.001).
Liu	2023	International consensus guidelines	N/A	Robotic liver resection is feasible among selected cirrhotic patients but evidence is still limited. Preliminary data showed that the robotic approach could bring similar benefits as laparoscopy.
Cipriani	2024	Retrospective comparative study	2534	The presence and severity of cirrhosis impact the difficulty of minimally invasive liver surgery as well as the outcome.
Zheng	2024	Retrospective comparative study	3675	Severity of CHILD score is associated with higher intraoperative and postoperative complications. Portal hypertension correlates with higher blood loss and increased need for transfusion.
Liu	2004	RCT	104	Abdominal drainage associated with increased morbidity rate (73% vs. 38%, *p* < 0.001) and length of stay. Collection rate was similar in both groups. Drainage was an independent risk factor for postoperative morbidity.
Fuster	2004	RCT	40	Intra‐abdominal closed drainage in cirrhotic patients undergoing liver resection is associated with reduced ascitic leakage and shorter hospital stay compared to the nondrainage group.
Ishizawa	2009	Case control study	203	A preoperative platelet count < 100 G/L and intraoperative blood loss > 1 L are associated with an increased risk of significant postoperative ascites.
Lee	2021	Prospective cohort	79	Preoperative edema assessed by bioelectrical impedance analysis is a significant predictor of postoperative ascites in cirrhotic patients undergoing liver surgery (OR 3.96 and *p* = 0.045).
Encephalopathy				
Gronbaek	2017	Post hoc cohort study	865	Cirrhotic patients using benzodiazepine (BZD) between 3 and 10 days have a significant risk of developing hepatic encephalopathy compared to common users (more than 4 weeks) and to a short exposition (1 or 2 days). Adjusted HR BZD use for 3–5 days versus non‐BZD: 4.9 (95% CI 1.5–15.8). Adjusted HR BZD use for 6–10 days versus non‐BZD: 5.5 (95% CI 1.6–19.0).
Kaiho	2005	RCT	84	Daikenchuto, an herbal medicine, decreases ammonium rate after liver surgery compared with lactulose (*p* < 0.05 at 1,7, and 10 days).
Shimada	2015	RCT	231	Postoperative use of daikenchuto accelerates time to first transit after hepatic resection and reduces systemic inflammation response among cirrhotic patients: Time to first bowel movement was 88.2 h (95% CI 74.0–94.1) versus 93.1 h (95% CI 83.3–99.4 and *p* = 0.046).
Hendry	2010	RCT	68	Routine use of magnesium hydroxide after hepatectomy significantly reduces time to first transit: Median 4 days (IQR 3–5) versus 5 days (IQR 4–6), *p* = 0.034.
Van Woerden	2022	RCT	82	Preoperative use of macrogol before liver surgery does not influence time to first transit nor functional recovery.
Jang	2012	Case control study	42	Chewing gum allows faster bowel recovery among patients with HCC undergoing liver surgery: Mean 77.3 h (SD 15) versus 87.4 h (SD 16.3), *p* = 0.026.
Anticoagulation				
Westerkamp	2009	Review	N/A	Cirrhosis and hepatectomy precipitate a dynamic coagulopathy, with complex interaction and balance between procoagulant and anticoagulant factors.
Tanner	2018	Prospective cohort	33	Using thromboelastogram, coagulation profile was similar between cirrhotic and noncirrhotic after liver surgery. Patients tend to have a hypercoagulable state.
Biancofiore	2019	Review	N/A	Viscoelastic tests (ROTEM) should be preferred over classical coagulation to guide transfusion in cirrhotic patients. Low CVP should be maintained during resection to decrease bleeding risk. Platelet transfusion should be considered to aim over 50,000/mm^3^.
Yoshikawa	2000	Controlled experimental study in animals	20	Administration of activated C protein prevents liver dysfunction after extended hepatectomy in rats.
Kaido	1999	Controlled experimental study in animals	N/A	Thrombomodulin infusion in cirrhotic hepatectomized rats improved the survival rate by preventing intrasinusoidal fibrin deposition and liver dysfunction.
Karunakaran	2022	MA	4238/8 studies	Use of pharmacological thromboprophylaxis after hepatectomy decreases VTE risk compared to mechanical thromboprophylaxis (2.5% vs. 5.3%; RR 0.5, and *p* = 0.03), without increasing bleeding risk.
Vivarelli	2010	Retrospective comparative study	229	Incidences of VTE and postoperative hemorrhage were similar after liver resection in cirrhotics patients with and without pharmacological thromboprophylaxis (VTE: 1.4% vs. 0.6%, *p* = 0.53; Hemorrhage: 3.2% vs. 1.4%, *p* = 0.38).
Wang	2018	RCT	233	Cirrhotic patients receiving pharmacological thromboprophylaxis demonstrated lower VTE prevalence compared to those without prophylaxis (0.9% vs. 13.8%, *p* < 0.05).
Yamashita	2014	Retrospective cohort	281	Thromboprophylaxis among cirrhotic patients after hepatic resection is associated with less portal venous thrombosis (10% vs. 2%; *p* = 0.04).
Aloia	2016	Expert consensus	N/A	Pharmacological thromboembolism prophylaxis in cirrhotic patients after hepatectomy is recommended if hemoglobin values are stable, platelet level > 100,000 mm^3^, and INR < 1.8.
Nutrition				
Umino	2022	Retrospective cohort	1272	The controlling nutritional status score (CONUT) has the best performance in prediction of morbidity after hepatectomy for HCC compared to 8 other nutrition/inflammatory scores (AUC 0.59, 95% CI 0.55–0.64, and *p* < 0.001).
Okubo	2021	Retrospective cohort	181	A high visceral‐to‐subcutaneous fat ratio is an independent predictor of poor long‐term prognosis in cirrhotic patients undergoing liver resection for HCC, being significantly associated with reduced OS (HR 2.7; 95% CI: 1.4–4.9; and *p* = 0.001) and faster progression to unresectable disease (HR 2.2; 95% CI 1.2–4.1; and *p* = 0.008).
Božin	2018	Retrospective cohort	38	ALBI and PALBI scores were identified as better prognostic factors for OS after hepatectomy for cirrhotic patients than MELD (high ALBI 23.1% higher RR of death. PALBI score 12.1% higher RR of death).
Okabayashi	2008	Retrospective comparative study	112	Oral supplementation with BCAA reduced the overall postoperative complication rate (18% vs. 44%, *p* = 0.019) and shortened the length of hospital stay (15 ± 5 vs. 22 ± 15 days, *p* < 0.05) in patients undergoing hepatectomy for HCC.
Ichikawa	2013	RCT	56	BCAA supplementation decreases the 30‐month recurrence rate after liver resection for HCC (cumulative risk of recurrence 28.5% vs. 55.7%), without any change for OS.
Chen	2015	MA	974 (11 studies)	BCAA supplementation decreases the ascites rate (RR = 0.55, 95% CI: 0.32–0.94, and *p* = 0.029) and edema rate (RR = 0.49, 95% CI: 0.26–0.95, and *p* = 0.035). Effect on mortality appears at long‐term follow‐up (3 years, RR = 0.80, 95% CI: 0.67–0.95, and *p* = 0.012).
Van Dijk	2023	MA	5184 (54 studies)	BCAA supplementation improved the event‐free survival (HR 0.61 and *p* = 0.008) and tends to improve OS (HR 0.58 and *p* = 0.05) in cirrhotic patients.
Zhang	2017	RCT	320	When compared to structolipid parenteral nutrition, N‐3 fatty acid‐based parenteral nutrition after hepatectomy for cirrhosis‐related liver cancer significantly reduces overall complications (29% vs. 50%, *p* < 0.001), infectious complications (10% vs. 19%, *p* = 0.014), and length of stay (10 vs. 13 days, *p* = 0.018).
Masuda	2013	Review	N/A	Oral nutrition support, including BCAA, symbiotics, and an immune enhancing diet, is recommended for cirrhotic patients undergoing liver surgery to prevent infectious complications.
Drainage				
Fuster	2004	RCT	N/A	Less ascites, LoS, and local complications in the drainage group.
Liu	2004	RCT	104	Morbidity: drainage: 73% versus no drainage: 38%, *p* < 0.001.
Kim	2014	RCT	200	Same morbidity but higher complications directly related to drainage.
Sun	2006	RCT	120	Wound complications higher with drainage (28% vs. 3%, *p* < 0.001).
Hajibandeh	2023	MA	1064 (7 studies)	Drainage increases overall (RR 1.37 and *p* < 0.001) and wound complications (RR 2.29 and *p* = 0.01).
Postoperative analgesia				
Siniscalchi	2016	Retrospective cohort	126	Epidurals: Lower MAP, more colloid infusions shorter ventilation time, and LoS. No epidural‐related complication.
Bateman	2013	Retrospective cohort	62450	7/62450 epidurals required surgery for epidural hematoma.
Taura	2003	RCT	104	Epidural morphine + small‐dose ketamine effective.
Fayed	2014	RCT	34	No difference in pain satisfaction between IV PCA and PCEA.
Yassen	2019	RCT	55	Less pain with TAP/RSB/PCA than fentanyl PCA alone.
Serag Eldin	2014	RCT	50	TAP with IV PCA decreased pain and fentanyl consumption.
Elshafie	2022	RCT	40	ESP blocks decreased opioid consumption versus conventional analgesia (*p* < 0.001).
Hidaka	2021	Prospective cohort	50	Acetaminophen safe in Child A patients.
Lim	2016	Retrospective cohort	101	PCA fentanyl and IV parecoxib effective in Child A patients.
Vascular filling				
Weinberg	2015	RCT	60	Similar base excess. More complications in the Hartmann solution group.
McFarlane	1994	RCT	30	Chloride concentration higher and increased base deficit with saline compared to Plasma‐Lyte.
Akanji	1989	Prospective cohort	12	Acetate infusion had similar metabolic effects in normal and diabetic subjects.
Akanji	1990	RCT	15	Infused acetate did not worsen glucose tolerance.
Sun	2014	Review	N/A	Tailored nutritional support and fluid therapy are recommended.
Barron	2004	SR	113 studies	Albumin had a lower rate of adverse events than other colloids.
Reinhart	2012	Consensus	N/A	Hydroxyl starch is not recommended for severe sepsis. Hyperoncotic solutions should be avoided for fluid resuscitation.
Salerno	2013	MA	288 (4 RCT)	Albumin prevented renal dysfunction and decreased mortality in patients with spontaneous bacterial peritonitis.
Wiedermann	2015	Review	N/A	Albumin has a nephroprotective potential.
Yang	2011	RCT	90	Hydroxyl starch or Ringer lactate can replace albumin in patients with HCC undergoing hepatectomy.
Liver failure				
Long	2023	Prospective cohort	266	High liver stiffness (> 9.5 kPa) was associated with liver failure.
Jehan	2024	PSM retrospective cohort	2129	Minor hepatectomy: Grade C liver failure: Open 2.3% versus MIS 0.9%, *p* = 0.03.
Wehrle	2023	Retrospective cohort	922	MIS lower rate of liver failure (HR 0.36).
Troisi	2021	PSM retrospective cohort	200	Benefits of MIS in patients without portal hypertension and in Child B7 patients.
Twaij	2014	SR and MA	420 (4 studies)	Laparoscopy safe in cirrhotic patients.
Famularo	2024	Retrospective cohort	500	Averaging ensemble model (machine learning): AUC 90.1% to predict liver failure with preoperative variables.
Chen	2012	Prospective cohort	190	Portal venous pressure and clinically significant PH predictors of liver failure (*p* < 0.001).
Wang	2022	Retrospective cohort	659	PH (OR 2.6), major hepatectomy (OR 4.2), and age (OR 1.1) predictors of liver failure.
Yoshino	2021	SR	28 studies	FLR volumes and portal hypertension significant predictors of liver failure.
Morandi	2023	Review	N/A	Better prediction when combining blood test scores, liver volumetry, and indocyanine green retention test.
Navarro	2020	Retrospective cohort	90	Platelet count and RLV/BW independent predictors of liver failure.
Prodeau	2019	Prospective cohort	343	Predictive model based on preoperative data.
Cucchetti	2017	Retrospective cohort	864	Predictors of liver failure and tumor recurrence.
Huang	2023	Retrospective cohort	146	Liver stiffness associated with liver failure.
Liang	2024	Prospective cohort	327	Creation of a predictive nomogram with preoperative and intraoperative details.
Vivarelli	2024	Retrospective cohort	130	Preservation of round ligament might prevent liver failure during MIS (12 vs. 30%, *p* = 0.011, and OR 6.8).

*Note:* N/A: data not reported or full‐text not available.

Abbreviations: BCAA, branched‐chain amino acids; BW, body weight; CVP, central venous pressure; ESP, erector spinae plane; FLR, future liver remnant; HBV, hepatitis B virus; HCC, hepatocellular carcinoma; INR, international normalized ratio; LoS, length of stay; MA, meta‐analysis; MAP, mean arterial pressure; MIS, minimally invasive surgery; OR, odds ratio; OS, overall survival; PC(E)A, patient controlled (epidural) analgesia; PH, portal hypertension; PSM, propensity‐score matching; RLV, remnant liver volume; RR, relative risk; SR, systematic review; TAP/RSB, transverseus abdominis plane/rectus sheath block; VTE, venous thromboembolic.

**TABLE 2:** Scores of quality and limitations of each included study.

First author	Quality score used to grade the study^a^	Score	Limitations
Ascites			
Zhang	STROBE	17/22	Temporal and selection bias.
Gines	N/A	N/A	N/A.
Zheng	Newcastle–Ottawa	8/8	Historical comparison group, sample size.
Chen	N/A	N/A	N/A.
Itoh	STROBE	16/22	Sample size, selection bias.
Coletta	AMSTAR	7/16	One RCT included.
Xu	Newcastle–Ottawa	8/8	No randomization, sample size.
Kanazawa	Newcastle–Ottawa	7/8	No randomization, monocentric, and small sample size, restrictive inclusion criteria.
Liu	N/A	N/A	N/A.
Cipriani	Newcastle–Ottawa	8/8	Heterogeneity due to the multicentric design.
Zheng	Newcastle–Ottawa	8/8	Heterogeneity due to the multicentric design.
Liu	Newcastle–Ottawa	7/8	Selection bias.
Fuster	Jadad score	3/5	No significant results for ascites leakage, sample size.
Ishizawa	Newcastle–Ottawa	7/8	Sample size.
Lee	Newcastle–Ottawa	7/8	Sample size.
Encephalopathy			
Gronbaek	Newcastle–Ottawa	8/8	No postoperative settings.
Kaiho	Jadad score	1/5	Group allocation, sample size.
Shimada	Jadad score	2/5	No blinding.
Hendry	Jadad score	3/5	Not only cirrhotic.
Van Woerden	Jadad score	3/5	Not only cirrhotic, optimal macrogol dose?.
Jang	Newcastle–Ottawa	5/8	Sample size, no data about compliance, and no specification about allocation.
Anticoagulation			
Westerkamp	N/A	N/A	N/A.
Tanner	Newcastle–Ottawa	7/8	Sample size.
Biancofiore	N/A	N/A	N/A.
Yoshikawa	N/A	N/A	Experimental study.
Kaido	N/A	N/A	Experimental study.
Karunakaran	AMSTAR	10/16	Intervention and population heterogeneity.
Vivarelli	Newcastle–Ottawa	6/8	Significant difference in groups (higher CHILD and MELD among patient w/o prophylaxis), low number of events.
Wang	Jadad score	1/5	Duration of follow‐up not mentioned.
Yamashita	Newcastle–Ottawa	7/8	Heterogeneity of groups, length of follow up, historical control group, and single center.
Aloia	N/A	N/A	N/A.
Nutrition			
Umino	Newcastle–Ottawa	7/8	Single center.
Okubo	Newcastle–Ottawa	8/8	Single center.
Božin	Newcastle–Ottawa	6/8	Single center, sample size, and only compensated CHILD A cirrhosis.
Okabayashi	Newcastle–Ottawa	6/8	Historical control group, single center.
Ichikawa	Jadad score	2/5	Sample size.
Chen	AMSTAR	7/16	Quality of included studies, only Asia, not only surgery, and heterogeneity of BCAA supplementation.
Van Dijk	AMSTAR	13/16	Not only surgery, heterogeneity of BCAA supplementation.
Zhang	Jadad score	5/5	Single center, short follow‐up.
Masuda	N/A	N/A	N/A.
Drainage			
Fuster	N/A	N/A	N/A.
Liu	Jadad score	3/5	Only 66% of patients had cirrhosis.
Kim	N/A	N/A	Mix of patients with and without chronic liver disease.
Sun	Jadad score	3/5	Not all patients had cirrhosis.
Hajibandeh	AMSTAR	14/16	Heterogeneity of included studies.
Postoperative analgesia			
Siniscalchi	Newcastle–Ottawa	7/8	56/183 were excluded because of preoperative coagulation disorders.
Bateman	STROBE	20/22	Only surgical treatments, attempts of epidural placement not collected.
Taura	Jadad score	3/5	Few data on surgical details.
Fayed	N/A	N/A	Low number of patients.
Yassen	Jadad score	4/5	Only open one‐sided blocks.
Serag Eldin	Jadad score	2/5	Low number of patients. No clear explanation of power calculation.
Elshafie	Jadad score	4/5	No limitation description. No clear explanation of power calculation.
Hidaka	N/A	N/A	Low number of patients.
Lim	STROBE	15/22	Few patient characteristics and no surgical details.
Vascular filling			
Weinberg	Jadad score	4/5	Low patient number, powered for base excess (main outcome), and wide confidence intervals in secondary outcomes.
McFarlane	Jadad score	0/5	Complications not evaluated.
Akanji	N/A	N/A	Low number of patients.
Akanji	N/A	N/A	Low number of patients.
Sun	N/A	N/A	No systematic review was performed.
Barron	AMSTAR	14/16	Inclusion of several study types (not only RCT).
Reinhart	N/A	N/A	Inclusions of studies on volume depletion in sepsis, ICU, cardiac surgery, head injury, and organ donors.
Salerno	AMSTAR	16/16	Only 4 studies included and 2 of them had small sample sizes.
Wiedermann	N/A	N/A	No systematic review was performed.
Yang	Jadad score	3/5	No blinding.
Liver failure			
Long	STROBE	19/22	Nomogram needs external validation.
Jehan	STROBE	16/22	Based on the NSQIP database.
Wehrle	STROBE	17/22	Based on the NSQIP database.
Troisi	STROBE	20/22	Retrospective design.
Twaij	AMSTAR	13/16	No RCT and small number of patients in each study.
Famularo	STROBE	19/22	Intraoperative details were not taken into consideration.
Chen	STROBE	20/22	Small sample size for subgroups and lack of comparison with other models.
Wang	STROBE	18/22	High number of patients with HBV infection.
Yoshino	AMSTAR	13/16	Only retrospective studies were included.
Morandi	AMSTAR	2/16	Narrative review, no systematic review was performed.
Navarro	STROBE	17/22	Retrospective and monocentric.
Prodeau	N/A	N/A	N/A.
Cucchetti	STROBE	18/22	Lack of external validation.
Huang	STROBE	18/22	Lack of external validation.
Liang	STROBE	19/22	The device to measure liver stiffness was not available in laparoscopy.
Vivarelli	STROBE	18/22	No randomization and no direct measure of the portal pressure.

*Note:* N/A: data not reported or full‐text not available.

Abbreviations: BCAA, branched chain amino acid; HBV, hepatitis B virus; ICU, intensive care unit; NSQIP, National Security Quality Improvement Program; RCT, randomized controlled trial.

^a^ As several study designs were included in this review, use of different quality scores was required. The quality of each included article was evaluated using the Jadad score (RCT) [22], the Newcastle–Ottawa scale (retrospective comparative studies) [23], the STROBE guidelines (cohort studies) [24], or the AMSTAR checklist (systematic reviews and meta‐analyses) [25]. References of these quality studies can be found in the reference list of the article.

**TABLE 3:** Summary of the 8 analyzed items, including their level of evidence.

Item	Summary	Evidence level
1. Prevention and management of ascites decompensation	Perioperative ascites should be addressed before surgery, as it is associated with increased morbidity. Implementing an ERAS program and utilizing laparoscopic techniques are recommended, as both measures help to reduce the risk of ascites development. Prophylactic abdominal drainage after surgery does not prevent ascites and is linked to higher morbidity.	Moderate
2. Hepatic encephalopathy (HE) prevention	High‐risk patients for hepatic encephalopathy (history of HE, severe cirrhosis, or high ASA class) require careful monitoring. Prompt management of complications, such as infections, acute kidney injury, constipation, and gastrointestinal bleeding, is crucial. Prolonged benzodiazepine use should be avoided. Although laxatives and some herbal medicines may reduce ammonia levels and hasten bowel movements, their impact on morbidity and HE remains limited and uncertain.	Low
3. Perioperioperative anticoagulation	Patients with cirrhosis are at an increased risk of thrombosis in the postoperative period due to a hypercoagulable state. Traditional coagulation tests should be interpreted with caution, and viscoelastic testing is preferred to assess the complex coagulation profile of cirrhotic patients. Both mechanical and pharmacological prophylaxis for venous thromboembolism should be considered after hepatectomy. Pharmacological prophylaxis is deemed safe if hemoglobin levels remain stable, platelet counts exceed 100′000/mm^3^, and INR is below 1.8.	Moderate
4. Perioperative nutrition	BCAA supplementation seems safe but positive effects after hepatectomy in cirrhotic patients are not clear yet. If parenteral nutrition is used, n‐3 fatty acid‐based regimen should be preferred.	Low
5. Prophylactic abdominal drainage	Routine drainage is not recommended for patients with chronic liver disease or cirrhosis if the surgical procedure is performed satisfactorily. Drainage‐related morbidity is common and does not effectively prevent intra‐abdominal collections. There is currently no specific data regarding the use of drainage in cases of hepatectomy involving biliary reconstructions.	High
6. Postoperative analgesia	For open hepatic resection, thoracic epidural analgesia (TEA) appears safe in selected cirrhotic patients with normal coagulation profiles. A single shot of epidural morphine combined with ketamine may be a suitable alternative for patients at risk of developing postoperative coagulation abnormalities. TEA is considered superior to intravenous patient‐controlled analgesia (IV PCA) for pain management in cirrhotic patients. However, adding an interfascial block to IV PCA is a viable alternative that reduces opioid consumption and postoperative nausea and vomiting (PONV).	Low
7. Vascular filling	In cirrhotic patients undergoing liver surgery, acetate‐buffered solutions are preferred for vascular filling over Hartmann's solution or 0.9% saline. When colloid solutions are required, albumin is recommended as the first choice over artificial colloids due to the risks of acute kidney injury and anaphylaxis associated with the latter. Preoperative optimization with albumin supplementation may be considered in selected cases.	High
8. Prevention of posthepatectomy liver failure	Risk of PHLF is increased in cirrhotic patients. Portal hypertension and laparoscopy seem to be the most important PHLF predictors. Preoperative assessment and treatment of portal hypertension with medications or interventional procedures are paramount to decrease PHLF and its associated mortality. Moreover, when feasible minimal invasive should be prioritized.	High

Abbreviations: ASA, American Society of Anesthesiologists; BCAA, branched‐chain amino acid; ERAS, Enhanced Recovery After Surgery; INR, international normalized ratio; PHLF, posthepatectomy liver failure.

### 3.1 Prevention and Management of Ascitic Decompensation

Preoperative ascites is a risk factor (RF) for postoperative morbidity [26]. In this context, preoperative measures, such as low‐sodium diet, diuretics, and paracentesis, should be considered [27]. Zheng et al. found that ERAS use compared to non‐ERAS already permitted to decrease the postoperative ascites rate in cirrhotic patients undergoing hepatectomy (4% vs. 15%, *p* = 0.046) [20]. Chen et al. found in a single‐center cross‐sectional study including 73 patients that indocyanine green retention at 15 min, tumor size > 10 cm, and red blood cell (RBC) transfusion were RF for postoperative ascites [28]. In a similar study from Japan, 14/73 patients with HCC developed refractory ascites and the only RF for ascites was segment VII resection (OR 11.8, 95% CI 1.8–76.7, and *p* = 0.009) due to extensive liver mobilization [29]. A recent meta‐analysis, including 1321 patients, showed that laparoscopy induced less postoperative complications than open surgery after liver resection in cirrhotic patients and a lower ascites rate (2.7% vs. 12.9%, *p* < 0.001) [30]. Similar results regarding postoperative ascites were found in a propensity score‐matched study comparing laparoscopic to open major liver surgery for HCC in cirrhotic patients (9.4% vs. 31.3% and *p* = 0.030) [31] and in a retrospective cohort study including 56 cirrhotic patients with HCC undergoing open and laparoscopic resections [32]. Regarding robotic surgery in cirrhotic patients, results remain scarce but preliminary data showed that robotic resection could bring similar benefits as laparoscopy [33]. The presence and severity of cirrhosis might affect the robotic resection difficulty [34, 35].

Regarding abdominal drainage, a RCT comparing intraoperative drainage versus no drainage in patients with chronic liver diseases (69 liver cirrhosis) showed a higher complication rate in the drainage group (73% vs. 38%, *p* < 0.001) but similar rates of ascites collection ≥ 2 cm on postoperative day (POD) 7 (12%, *p* > 0.05) [36]. Another RCT comparing drainage to no drainage after liver surgery in cirrhotic patients showed less ascites and shorter LoS in the drainage group [37].

In a case‐control study of patients with HCC, Ishizawa et al. found that preoperative platelet count < 100 G/L and intraoperative blood loss > 1 L were RF to develop postoperative ascites [38]. Diuretics and fresh‐frozen plasma were effective in treating these patients. In a prospective study (*n* = 79), Lee et al. found that a high preoperative edema index (extracellular water/total body water) measured using bioelectrical impedance was a RF for postoperative ascites in cirrhotic patients (OR 3.96 and *p* = 0.045) [39].

The evidence level of included articles was moderate.

### 3.2 Hepatic Encephalopathy (HE) Prevention

Many conditions, such as constipation, infection, or gastrointestinal bleeding, can precipitate HE [40]. In a study on 865 cirrhotic patients using data of 3 large prospective trials, HE was associated with benzodiazepine use [41]. One RCT evaluated the impact of daikenchuto, a traditional herbal medicine, on postoperative serum ammonia levels in hepatectomized patients compared with lactulose [42]. They found that daikenchuto significantly reduced ammonia levels and delayed flatulence. Another multicentric RCT investigated the efficacy of daikenchuto following hepatectomy among 231 patients with Child A/B and provided evidence that daikenchuto accelerated the time‐to‐first bowel movement and reduced systemic inflammatory response [43]. Complication rates were similar in both studies. Hendry et al. showed in a RCT that magnesium hydroxide after hepatectomy shortened time‐to‐first transit [44]. A more recent and well‐conducted RCT showed no advantage of preoperative macrogol before liver surgery on postoperative morbidity and time‐to‐first transit [45]. Proportion of cirrhotic patients and subgroup analyses were not specified in both studies. In a case‐control study on 42 patients with HCC, chewing gum demonstrated faster bowel function recovery [46].

Included articles had low evidence level.

### 3.3 Perioperative Anticoagulation

New data have shown that several changes favor hemostasis and other increase the bleeding risk in cirrhotic patients (procoagulant effect: increased von Willenbrandt factor and anticoagulant effect: thrombocytopenia, platelet dysfunction) [47]. A 2018 prospective study found that cirrhotic patients had similar coagulation profiles as noncirrhotic patients after liver surgery, but patients were often in a hypercoagulable state [48].

Furthermore, classic prothrombin and activated partial thromboplastin times should not be used in cirrhotic patients to guide transfusion. Viscoelastic tests, such as ROTEM, should be preferred [49].

Some studies in cirrhotic rats have shown that injection of activated protein C or soluble thrombomodulin after extended hepatectomy permitted to decrease the postoperative liver dysfunction [50, 51], but no data currently exist in human.

Preoperative anemia should be diagnosed and corrected preoperatively if possible. In case of significant intraoperative bleeding, platelets in addition to RBC should be administered and a thrombocyte threshold of 50,000/mm^3^ should be aimed [49]. As in other hepatectomy, low central venous pressure during resection should be maintained to decrease the bleeding risk [49].

A recent meta‐analysis of eight studies, including 4238 patients, recommended pharmacological and mechanical thromboprophylaxis to decrease the risk of posthepatectomy thromboembolic events [52]. Of note, three studies included only cirrhotic patients and were in favor of using perioperative mixed thromboprophylaxis [53, 54, 55]. A conference consensus, including HPB surgeons and hematology specialists, published in 2016 suggested to use venous thromboembolism prophylaxis in cirrhotic patients after hepatectomy if hemoglobin values are stable, platelet level > 100,000 mm^3^, and INR < 1.8 [56].

The evidence level was judged as moderate for this item.

### 3.4 Perioperative Nutrition

The controlling nutritional status score (including albumin, lymphocyte, and total cholesterol) permits to stratify the risk of complications after hepatectomy in cirrhotic patients [57]. Moreover, the ratio visceral fat/subcutaneous tissue was found to be predictive of overall survival (OS) in operated cirrhotic patients with HCC (HR 2.65 and *p* = 0.001) [58]. ALBI and PALBI scores were also found to be prognostic factors of OS after hepatectomy among cirrhotic patients on the contrary of the MELD score [59].

Okabayashi et al. showed that branched‐chain amino acid (BCAA) supplementation permitted to decrease the overall complication rate in patients undergoing hepatectomy for HCC (18% vs. 44%, *p* = 0.01) [60]. A RCT on 56 patients found that oral BCAA supplementation decreased the 30‐month recurrence rate after liver resection for HCC but OS was similar [61]. In a meta‐analysis of 11 nonrandomized studies from Asia with variable BCAA supplementation times (range: 0.5–12 months), the authors found that BCAA supplementation was safe and decreased the ascites rate but that effect on mortality was not confirmed [62]. A recent meta‐analysis of 54 studies on the role of BCAA supplementation showed that BCAA improved the event‐free survival (HR 0.61 and *p* = 0.008) and OS (HR 0.58 and *p* = 0.05) in cirrhotic patients [63].

A RCT by Zhang compared an n‐3 fatty acid‐based parenteral nutrition to structolipid parenteral nutrition for 5 days after hepatectomy for patients with cirrhosis‐induced liver cancer [64]. The 157 patients of the n‐3 fatty acid‐based parenteral nutrition had fewer overall complications (29% vs. 50%, *p* < 0.001), fewer infectious complications (10% vs. 19%, *p* = 0.014), and shorter mean LoS (10 vs. 13 days, *p* = 0.018).

In a 2013 review, Masuda et al. recommended oral nutrition support, including BCAA, symbiotics, and an immune enhancing diet, for cirrhotic patients undergoing liver surgery to prevent perioperative infectious complications [65].

The level of evidence of the included articles was low.

### 3.5 Prophylactic Abdominal Drainage

One RCT published in 2004 specifically addressed this issue among 40 cirrhotic patients with HCC who were randomized into drainage and no drainage [37]. No difference was found in morbimortality between both groups. However, the authors reported a longer LoS among the nondrainage group (10 ± 3 vs. 14 ± 4 days, *p* = 0.05). Interpretation of those results should be cautious, as patient with clinically relevant portal hypertension (hepatic venous pressure gradient > 10 mmHg), which is a known RF for postoperative ascites, were more represented in the nondrainage group. Another well‐designed RCT evaluated abdominal drainage among patients with chronic liver disease (66% of cirrhotic patients) after hepatectomy (*n* = 104) [36]. The authors found opposite results, with higher postoperative morbidity (mainly wound infections) as well as longer LoS in the drainage group. Moreover, the authors identified prophylactic drainage as an independent RF for postoperative morbidity. Interestingly, they systematically looked for postoperative site collection with ultrasonography on POD7 and reported no differences concerning the presence and quality of collections among both groups. A more recent RCT published in 2014 compared drainage versus nondrainage after hepatectomy among 200 patients and found no difference in postoperative morbidity [66]. The authors performed subgroup analysis for chronic hepatitis and cirrhotic patients (*n* = 108) and results became significant, with more complication in the drainage group (1 vs. 7, *p* = 0.027). Therefore, the authors did not recommend systematic drainage. Another RCT with cirrhotic patients accounting for 67% of the population found similar results: higher rate of surgical complications, particularly wound infections and ascites leakages [67]. A recent systematic review and meta‐analysis by Hajibandeh et al. reviewed the results of 7 RCT including a total of 1064 patients [68]. The authors concluded that prophylactic abdominal drainage increased the overall and wound‐related complications, without decreasing the risk of intra‐abdominal collections. The rate of cirrhotic patients in the pooled population was almost 50% and similar in both groups.

The level of evidence was judged as high.

### 3.6 Postoperative Analgesia

A retrospective study compared thoracic epidural anesthesia (TEA) to PCA among cirrhotic patients after open liver surgery [69]. Patients in the epidural group had shorter mechanical ventilation time and LoS. Moreover, no patient in the epidural group had complication related to the positioning nor the removal of the catheter. However, one third of patients were excluded due to contraindication to the placement of epidural catheter (platelet count < 100,000/L or INR > 1.5). Epidural complication requiring surgical treatment is a rare event (1/4330 epidural placements) [70].

One RCT evaluated a single epidural bolus of morphine associated with ketamine given after open hepatic resection in cirrhotic patients [71]. The authors demonstrated that when compared with epidural morphine alone, adjunction of ketamine enhanced analgesic effect of morphine, lengthened pain relief (27.2 ± 8 h vs. 16.4 ± 10 h, *p* < 0.05), and significantly reduced the need for complementary doses of analgesia (7% vs. 100%, *p* < 0.05) without side effects. Fayed et al. published in 2014 a RCT comparing IV PCA with fentanyl to patient‐controlled epidural analgesia (PCEA) among 34 cirrhotic patients after hepatic resection [72]. However, average pain score as well as pain upon movement and coughing were lower in the PCEA group. The authors reported no complication due to epidural catheter removal.

Several RCT have evaluated safety and efficacity of transversus abdominis plane (TAP) block, rectus sheath space block (RSB), or erector spinae plane block (ESPB) administered as a single shot or continuously through catheters. Yassen et al. published a RCT evaluating fentanyl PCA with and without repeated boluses of bupivacaine through catheter placed in the TAP and rectal sheath spaces among 55 cirrhotic patients undergoing open liver resection [73]. They achieved better pain control during movement and reduced total fentanyl consumption as well as postoperative nausea and vomiting (PONV). Those results are supported by another RCT evaluating the effect of TAP in adjunction to intravenous fentanyl PCA on 50 cirrhotic patients after open liver resection [74]. Combining TAP with IV PCA improved postoperative pain management and reduced total fentanyl consumption. Finally, one RCT published in 2022 assessed ESPB with opioid‐free anesthesia compared with conventional balanced anesthesia with opioid among 43 cirrhotic patients undergoing liver resection [75]. The authors found convincing evidence with reduced opioid consumption, delay to first request for analgesia, and reduced PONV. Catheters should nevertheless be placed with caution with regards to collateral abdominal wall veins.

One recent Japanese study showed the safety of acetaminophen (15 mg/kg) in Child A patients undergoing liver resection [76]. One retrospective study compared the effect of IV PCA fentanyl with a single dose of parecoxib between patients with healthy livers and Child A patients undergoing major liver resection [77]. The pain control with PCA of fentanyl and with single dose of parecoxib was better in the group with liver disease.

For this item, the evidence level of the included articles was low.

### 3.7 Vascular Filling

A multicentric RCT compared two frequent crystalloids used during major liver resection: Hartmann solution (HS) and Plasma‐Lyte‐148 (acetate buffered solution) [78]. Patients (no precision if cirrhotic patients were included) treated with Plasma‐Lyte‐148 had better biochemical and hematological profiles (electrolyte balance, coagulation status, and acid‐base homeostasis). Interestingly, the HS group had higher blood loss and overall complications. Acetate‐based solutions have also shown benefits compared to 0.9% saline during hepatic resection, with less hyperchloremic acidosis [79]. Finally, acetate solutions can act as a substitute to fat and can prevent malnutrition, without any influence on glucose homeostasis, which can be dysregulated after liver surgery [80, 81].

Some authors argued that preoperative albumin supplementation in selected cases (cirrhosis, multisegmental hepatectomy, or clear hypoalbuminemia) could reduce postoperative complications after liver resection [82]. Indeed, albumin represents an attractive colloid in hepatic resection because of the maintenance of osmotic pressure, absence of anaphylaxis and side effect, safety (absence of nephrotoxicity), and effectiveness (reduction of renal morbidity among cirrhotic) [83, 84, 85, 86]. One RCT among cirrhotic patients undergoing hepatic resection for HCC compared the effect of 20% human albumin (HA), artificial colloid (HES), and crystalloid [87]. More fluid was needed in the crystalloid group and subsequently morbidity was higher. The authors found no differences concerning hemodynamics, urine outputs nor blood product use between all three groups. Interestingly acute inflammatory response was lower in the artificial colloid group.

The level of evidence was high for this item.

### 3.8 Prevention of Postoperative Liver Failure

Cirrhotic patients are more at risk of posthepatectomy liver failure (PHLF) and the risk of PHLF increases with the Child–Pugh grade [88]. Several studies, including an analysis of the NSQIP database and several meta‐analyses, showed that minimally invasive surgery has lower risk of developing PHLF than open surgery and should be performed if feasible [89, 90, 91, 92]. A recent machine learning and radiomics analysis found that macrovascular invasion, major resection, and high MELD score, in addition to cirrhosis, were predictors of PHLF in patients with HCC [93]. Chen et al. showed in a prospective study (*n* = 190 cirrhotic patients) that the severity of portal hypertension and the neutrophil‐to‐lymphocyte ratio were independent factors of PHLF after hepatectomy, highlighting the importance of reducing the portal pressure preoperatively with drugs or interventional procedures [94]. The importance of portal hypertension on PHLF was corroborated by several other studies [88, 95, 96]. Regarding PHLF prediction, a systematic review recommended to combine biologic scores, liver volumetry, and indocyanine green retention test to obtain a more precise estimation [97]. Volume and function of liver remnant were associated with PHLF in several studies [88, 96, 98]. Prodeau et al. found in a French multicentric cohort study that, in addition to liver remnant volume, preoperative platelet count and intention to perform laparoscopy were preoperative predictors of PHLF in cirrhotic patients undergoing hepatectomy [99]. In a bicentric study of Child A cirrhotic patients, hepatitis C infection was found as risk for PHLF death following hepatectomy [100]. Preoperative and intraoperative assessments of the liver stiffness by elastography have been described as a potential tool to predict the postoperative risk of PHLF [101, 102]. A retrospective Italian study (*n* = 130) showed that nondivision of the round ligament during laparoscopic hepatectomy was associated with less PHLF [103].

Evidence level was judged as high for this item.

## 4 Discussion

This systematic review synthesized the best available evidence regarding several specific items that need to be considered in cirrhotic patients undergoing liver surgery.

Preoperative ascites was found as a RF for postoperative morbidity. Therefore, presence of ascites should be managed prior to surgery using measures such as a low‐sodium diet, diuretics, and paracentesis. Laparoscopy should be preferred to open surgery as it offers less complication, including lower postoperative ascites rate.

Particular attention should be given to identify patients at high‐risk of HE (HE history, high ASA class, and cirrhosis degree). Early recognition and management of complications, such as infection, acute kidney injury, constipation, and gastrointestinal bleeding, are essential. Prolonged benzodiazepine use should be avoided. Postoperative administration of laxatives and certain herbal medicines have been shown to shorten the time‐to‐first bowel movement and reduce ammonia levels after hepatectomy. However, their effects on morbidity remain minor, and their influence on HE is still uncertain.

Liver cirrhosis induces a perioperative hypercoagulable state following hepatectomy. Therefore, mechanical and pharmacological venous thromboembolism prophylaxes are recommended postoperatively if hemoglobin count is stable, platelet level is correct, and coagulation tests are within normal range.

Cirrhotic patients often have protein malnutrition. BCAA supplementation seems safe, but positive effects after hepatectomy in cirrhotic patients are not clear yet. If parenteral nutrition is used, n‐3 fatty acid‐based regimen should be preferred.

Systematic drainage among patients with cirrhosis is not recommended if the surgical procedure is satisfactory. Drainage site related morbidity is frequent and does not prevent intra‐abdominal collection. No specific data on drainage in case of hepatectomy with biliary reconstructions were found.

For open hepatic resection, TEA seems safe in selected cirrhotic patients without abnormal coagulation profiles. Single shot of epidural morphine and ketamine might be a suitable alternative among patients with RF of developing postoperative abnormal coagulation profile. TEA seems superior to IV PCA in pain management for cirrhotic patients. However, adjunction of interfascial block to IV PCA is a good alternative to decrease opioid consumption and PONV and offers good pain control. The ideal infusion regimen for cirrhotic patients as well as analgesic adjuvants are not yet determined.

Vascular filling with acetate‐buffered solutions should be preferred over HS or 0.9% saline. When colloid solution is indicated, albumin should be the first choice over artificial colloid solution because of the risk of acute kidney injury and anaphylaxis. Preoperative optimization with albumin supplementation could be proposed in selected cases.

Risk of PHLF is increased in cirrhotic patients. Portal hypertension and laparoscopy seem to be the most important predictors of PHLF. Preoperative assessment and treatment of portal hypertension with medications or interventional procedures are paramount to decrease PHLF and its associated mortality. Moreover, when feasible, minimally invasive approach should be prioritized.

In conclusion, the management of cirrhotic patients undergoing liver surgery presents unique challenges that require specific perioperative strategies. The presented evidence lays the cornerstones for future specific perioperative recommendations and guidelines for cirrhotic patients undergoing liver surgery.

## Author Contributions

**Gaetan‐Romain Joliat:** conceptualization, methodology, data curation, formal analysis, project administration, writing – original draft, writing – review and editing. **Constant Delabays:** conceptualization, methodology, data curation, formal analysis, project administration, writing – original draft, writing – review and editing. **Emilie Uldry:** validation, supervision, formal analysis, writing – review and editing. **Valerie Addor:** validation, formal analysis, supervision, writing – review and editing. **David Fuks:** validation, formal analysis, supervision, writing – review and editing. **Emmanuel Melloul:** conceptualization, methodology, validation, formal analysis, supervision, project administration, writing – review and editing.

## Conflicts of Interest

The authors declare no conflicts of interest.

## Supporting information

Supporting Information S1

## Data Availability

The data that support the findings of this study are available from the corresponding author upon reasonable request.
