# Supplementary material for: Specific Items of Enhanced Recovery After Surgery for Liver Surgery in Cirrhotic Patients: A Systematic Review
Source: World J Surg. 2025 Jun 23;49(8):2125–43. doi: 10.1002/wjs.12677 (PMC12338391; doi:10.1002/wjs.12677)
Supplement: Supplementary file 1 — Supporting Information S1 [file WJS-49-2125-s001.docx]

**Supplementary material**

*MEDLINE/PubMed equations used for search*

For prevention and management of perioperative ascites

(“Hepatectomy” [Mesh] OR “Liver Surgery”) AND (“Liver Cirrhosis” [Mesh] OR “Cirrhosis”) AND “Ascites” [Mesh]

For encephalopathy prevention

(“Hepatectomy” [Mesh] OR “Liver Surgery”) AND (“Liver Cirrhosis” [Mesh] OR “Cirrhosis”) AND (“Hepatic Encephalopathy” [Mesh] OR “Encephalopathy”)

For perioperative anticoagulation

(“Hepatectomy” [Mesh] OR “Liver Surgery”) AND (“Liver Cirrhosis” [Mesh] OR “Cirrhosis”) AND (“Anticoagulants” [Mesh] OR “Anticoagulation” OR “Thrombosis Prevention” OR “Thrombosis Prophylaxis”)

For perioperative nutrition

(“Hepatectomy” [Mesh] OR “Liver Surgery”) AND (“Liver Cirrhosis” [Mesh] OR “Cirrhosis”) AND (“Diet, Food, and Nutrition” [Mesh] OR “Nutrition”)

For prophylactic abdominal drainage

(“Hepatectomy” [Mesh] OR “Liver Surgery”) AND (“Liver Cirrhosis” [Mesh] OR “Cirrhosis”) AND “Drainage” [Mesh]

For postoperative analgesia

(“Hepatectomy” [Mesh] OR “Liver Surgery”) AND (“Liver Cirrhosis” [Mesh] OR “Cirrhosis”) AND (“Analgesia” [Mesh] OR “Anesthesia and Analgesia” [Mesh])

For vascular filling

(“Hepatectomy” [Mesh] OR “Liver Surgery”) AND (“Liver Cirrhosis” [Mesh] OR “Cirrhosis”) AND (“Perfusion” [Mesh] OR “Vascular filling” OR “Intravenous Infusion” [Mesh])

For prevention of liver failure

(“Hepatectomy” [Mesh] OR “Liver Surgery”) AND (“Liver Cirrhosis” [Mesh] OR “Cirrhosis”) AND (“Liver failure” [Mesh] OR “Liver insufficiency” OR “Liver failure, Acute” [Mesh] OR “Hepatic Insufficiency” [Mesh])
